# Supplementary material for: Artificial intelligence in ovarian cancer histopathology: a systematic review
Source: NPJ Precis Oncol. 2023 Aug 31;7:83. doi: 10.1038/s41698-023-00432-6 (PMC10471607; doi:10.1038/s41698-023-00432-6)
Supplement: Supplementary file 1 — Supplemental Material [file 41698_2023_432_MOESM1_ESM.pdf]

Supplementary Information - Artificial Intelligence in Ovarian Cancer Histopathology: A Systematic Review

Contents

Supplementary Note 1 - Literature searches **2**

Supplementary Table 1 - Data extraction fields **4**

Supplementary Table 2 - PRISMA 2020 abstract checklist **5**

Supplementary Table 3 - PRISMA 2020 full checklist **6**

Supplementary Note 1 - Literature searches

Literature searches for all databases are shown here, with any text which is not directly input to the search bar in **bold** font. These searches are each a combination of three aspects - artificial intelligence, ovarian cancer, and histopathology. No filters were applied, and all options were left on their default settings. The wildcard character, *, was used to search for multiple versions of the same word, for example, “patholog*” searches for all of “pathology”, “pathologist”, “pathologists”, and “pathological”.

PubMed
(“Machine Learning”[Mesh] OR “Artificial Intelligence”[Mesh] OR “Neural Networks, Computer”[Mesh] OR “support vector machine”[MeSH] OR “Deep Learning”[Mesh] OR “diagnosis, computer-assisted ”[Mesh] OR “Machine learn∗” OR “Artificial Intelligen∗” OR (ML[Title/Abstract] NOT (μgml[Title/ Abstract] OR μ/ml[Title/Abstract] OR mgml[Title/Abstract] OR pgml[Title/Abstract] OR ngml[Title/Abstract] OR uiml[Title/Abstract] OR iuml[Title/Abstract] OR miuml[Title/Abstract] OR muiml[Title/Abstract] OR uml[Title/Abstract] OR gml[Title/Abstract] OR mlkg[Title/Abstract] OR milliliter∗[Title/Abstract])) OR AI[Title/Abstract] OR “Computer Vision” OR “Neural network∗” OR “Deep Network∗” OR “Computer-aided Diagnosis” OR “Computer aided Diagnosis” OR Perceptron∗ OR “Convolutional Network∗” OR “Recurrent Network∗” OR “Graph Network∗” OR “Deep Learn∗” OR “Deep-Learn∗” OR Backprop∗ OR “support vector∗” OR ensemble∗ OR “random forest∗” OR “nearest neighbor∗” OR “nearest neighbour∗” OR “k-nearest neighbor∗” OR “k-nearest neighbour∗” OR “Gradient boost∗” OR “XGBoost∗” OR “segmentation” OR “instance learning” OR “multi-instance learning” OR “Active Learning”)

AND (((ovar∗ OR fallopian) AND (cancer∗ OR mass∗ OR carcinoma∗ OR tumour∗ OR tumor∗ OR neoplasm∗ OR malignan∗ OR “carcinoma”[Mesh] OR “neoplasms”[Mesh])) OR “Ovarian Neoplasms”[Mesh] OR “peritoneal cancer” OR “peritoneal carcinoma” OR “peritoneal tumo∗”)

AND ((digit∗ AND patholog∗) OR “computational patholog∗” OR “tissue microarray∗” OR histopath∗ OR histolog∗ OR “Whole Slide Imag∗” OR “Tissue slide∗” OR “pathology slide∗” OR “pathology image∗” OR Immunohistochem∗ OR ((Haematoxylin OR Hematoxylin) AND Eosin) OR Histology[Mesh])

Scopus
TITLE-ABS-KEY(“Machine learn∗” OR “Artificial Intelligen∗” OR (“ML” AND NOT “∗ μ ml” AND NOT “∗g ml” AND NOT “∗ui ml” AND NOT “∗UI ml” AND NOT “∗iu ml” AND NOT “∗u ml” AND NOT “∗g ml” AND NOT “∗ml kg” AND NOT milliliter∗) OR AI OR “Computer Vision” OR “Neural network∗” OR “Deep Network∗” OR “Computer-aided Diagnosis” OR “Computer aided Diagnosis” OR Perceptron∗ OR “Convolutional Network∗” OR “Recurrent Network∗” OR “Graph Network∗” OR “Deep Learn∗” OR “Deep-Learn∗” OR Backprop∗ OR “support vector∗” OR ensemble∗ OR “random forest∗” OR “nearest neighbor∗” OR “nearest neighbour∗” OR “k-nearest neighbor∗” OR “k-nearest neighbour∗” OR “Gradient boost∗” OR “XGBoost∗” OR “segmentation” OR “instance learning” OR “multi-instance learning” OR “Active Learning”)

AND TITLE-ABS-KEY(((ovar∗ OR fallopian) AND (cancer∗ OR mass∗ OR carcinoma∗ OR tumour∗ OR tumor∗ OR neoplasm∗ OR malignan∗)) OR “peritoneal cancer” OR “peritoneal carcinoma” OR “peritoneal tumo∗”)

AND TITLE-ABS-KEY((digit∗ AND patholog∗) OR “computational patholog∗” OR “tissue microarray∗” OR histopath∗ OR histolog∗ OR “Whole Slide Imag∗” OR “Tissue slide∗” OR “pathology slide∗” OR “pathology image∗” OR Immunohistochem∗ OR ((Haematoxylin OR Hematoxylin) AND Eosin))

Web of Science
(ALL=(“Machine learn∗” OR “Artificial Intelligen∗” OR “Computer Vision” OR “Neural network∗” OR “Deep Network∗” OR “Computer-aided Diagnosis” OR “Computer aided Diagnosis” OR Perceptron∗ OR “Convolutional Network∗” OR “Recurrent Network∗” OR “Graph Network∗” OR “Deep Learn∗” OR “Deep-Learn∗” OR Backprop∗ OR “support vector∗” OR ensemble∗ OR “random forest∗” OR “nearest neighbor∗” OR “nearest neighbour∗” OR “k-nearest neighbor∗” OR “k-nearest neighbour∗” OR “Gradient boost∗” OR “XGBoost∗” OR “segmentation” OR “instance learning” OR “multi-instance

learning” OR “Active Learning”) OR TS=(AI OR (“ML” NOT (“∗ μ ml” OR “∗g ml” OR “∗ui ml” OR “∗UI ml” OR “∗iu ml” OR “∗u ml” OR “∗g ml” OR “∗ml kg” OR milliliter∗))))

AND ALL=(((ovar∗ OR fallopian) AND (cancer∗ OR mass∗ OR carcinoma∗ OR tumour∗ OR tumor∗ OR neoplasm∗ OR malignan∗)) OR “peritoneal cancer” OR “peritoneal carcinoma” OR “peritoneal tumo∗”)

AND ALL=((digit∗ AND patholog∗) OR “computational patholog∗” OR “tissue microarray∗” OR histopath∗ OR histolog∗ OR “Whole Slide Imag∗” OR “Tissue slide∗” OR “pathology slide∗” OR “pathology image∗” OR Immunohistochem∗ OR ((Haematoxylin OR Hematoxylin) AND Eosin))

Cochrane Central Register of Controlled Trials

Search #1:

All text: (“Machine learn∗” OR “Artificial Intelligen∗” OR “Computer Vision” OR “Neural network∗” OR “Deep Network∗” OR “Computer-aided Diagnosis” OR “Computer aided Diagnosis” OR Perceptron∗ OR “Convolutional Network∗” OR “Recurrent Network∗” OR “Graph Network∗” OR “Deep Learn∗” OR “Deep-Learn∗” OR Backprop∗ OR “support vector∗” OR ensemble∗ OR “random forest∗” OR “nearest neighbor∗” OR “nearest neighbour∗” OR “k-nearest neighbor∗” OR “k-nearest neighbour∗” OR “Gradient boost∗” OR “XGBoost∗” OR “segmentation” OR “instance learning” OR “multi-instance learning” OR “Active Learning”)

Search #2:

Title-Abstract-Keyword: (“AI” OR (“ML” NOT (“∗ μ ml” OR “∗g ml” OR “∗ui ml” OR “∗UI ml” OR “∗iu ml” OR “∗u ml” OR “∗g ml” OR “∗ml kg” OR milliliter∗))) in Title Abstract Keyword

Search #3:

All text: (((ovar∗ OR fallopian) AND (cancer∗ OR mass∗ OR carcinoma∗ OR tumour∗ OR tumor∗ OR neoplasm∗ OR malignan∗)) OR “peritoneal cancer” OR “peritoneal carcinoma” OR “peritoneal tumo∗”)

AND ((digit∗ AND patholog∗) OR “computational patholog∗” OR “tissue microarray∗” OR histopath∗ OR histolog∗ OR “Whole Slide Imag∗” OR “Tissue slide∗” OR “pathology slide∗” OR “pathology image∗” OR Immunohistochem∗ OR ((Haematoxylin OR Hematoxylin) AND Eosin))

Final search:

(#1 OR #2) AND #3

WHO-ICTRP

((“Machine learn∗” OR “Artificial Intelligen∗” OR “Computer Vision” OR “Neural network∗” OR “Deep Network∗” OR “Computer-aided Diagnosis” OR “Computer aided Diagnosis” OR Perceptron∗ OR “Convolutional Network∗” OR “Recurrent Network∗” OR “Graph Network∗” OR “Deep Learn∗” OR “Deep-Learn∗” OR Backprop∗ OR “support vector∗” OR ensemble∗ OR “random forest∗” OR “nearest neighbor∗” OR “nearest neighbour∗” OR “k-nearest neighbor∗” OR “k-nearest neighbour∗” OR “Gradient boost∗” OR “XGBoost∗” OR “segmentation” OR “instance learning” OR “multi-instance learning” OR “Active Learning”) OR (“AI” OR (“ML” NOT (“μ/ml” OR “g/ml” OR “ui/ml” OR “UI/ml” OR “iu/ml” OR “u/ml” OR “g/ml” OR “ml/kg” OR milliliter∗))))

AND (((ovar∗ OR fallopian) AND (cancer∗ OR mass∗ OR carcinoma∗ OR tumour∗ OR tumor∗ OR neoplasm∗ OR malignan∗)) OR “peritoneal cancer” OR “peritoneal carcinoma” OR “peritoneal tumo∗”)

AND ((digit∗ AND patholog∗) OR “computational patholog∗” OR “tissue microarray∗” OR histopath∗ OR histolog∗ OR “Whole Slide Imag∗” OR “Tissue slide∗” OR “pathology slide∗” OR “pathology image∗” OR Immunohistochem∗ OR ((Haematoxylin OR Hematoxylin) AND Eosin))

Supplementary Table 1 - Data extraction fields

Category	Fields
Overview	Internal ID. Lead author. Year. Conference/Journal name.
Data	Number of development images. Total number of images. Type of samples. FFPE/Frozen. Size of images. Tissue of origin. Number of development patients. Total number of patients. Number of data collection centres. Type of stain. Number of stainers. Scanners. Number of scanner types. Number of tissue processing centes. Data origin countries. Number of pathologists for data labelling. Online dataset. Prospective/retrospective. Clinical/research tissue. Data annotation. Maximum magnification available. Supplementary datatypes. Data exclusion reasons. Number of images excluded. Other cancer types included.
Methods	Outcome. Outcome measure/classes. Outcome standards/definition. Magnifications used. Patch sizes. Patches per image. Task type. Feature extraction type. Feature extractors. AI in main method. Other AI methods. Optimiser. Number of external validations. Differences to external validation set. Total external validation images. Number of cross-validation folds. Number of non-novel methods compared. Number of GPUs. Type of GPUs.
Results	Internal test accuracy, error bounds. AUC, error bounds. Sensitivity/specificity, error bounds. Other metric 1, error bounds. Other metric 2, error bounds. Other metric 3, error bounds. External training type. External test accuracy, error bounds. AUC, error bounds. Sensitivity/specificity, error bounds. Other metric 1, error bounds. Other metric 2, error bounds. Other metric 3, error bounds. Type of error bounds. Model training time. Visualisation type.
Miscellaneous	Code availability. Data availability. Notes

Summary of the fields used for data extraction. The full form is available at `www.github.com/scjjb/OvCaReview`.

Supplementary Table 2 - PRISMA 2020 abstract checklist

PRISMA 2020 for Abstracts Checklist

Section and Topic	Item #	Checklist item	Reported (Yes/No)
**TITLE**			
Title	1	Identify the report as a systematic review.	Yes
**BACKGROUND**			
Objectives	2	Provide an explicit statement of the main objective(s) or question(s) the review addresses.	Yes
**METHODS**			
Eligibility criteria	3	Specify the inclusion and exclusion criteria for the review.	Yes
Information sources	4	Specify the information sources (e.g. databases, registers) used to identify studies and the date when each was last searched.	Yes
Risk of bias	5	Specify the methods used to assess risk of bias in the included studies.	Yes
Synthesis of results	6	Specify the methods used to present and synthesise results.	Yes
**RESULTS**			
Included studies	7	Give the total number of included studies and participants and summarise relevant characteristics of studies.	Yes
Synthesis of results	8	Present results for main outcomes, preferably indicating the number of included studies and participants for each. If meta-analysis was done, report the summary estimate and confidence/credible interval. If comparing groups, indicate the direction of the effect (i.e. which group is favoured).	Yes
**DISCUSSION**			
Limitations of evidence	9	Provide a brief summary of the limitations of the evidence included in the review (e.g. study risk of bias, inconsistency and imprecision).	Yes
Interpretation	10	Provide a general interpretation of the results and important implications.	Yes
**OTHER**			
Funding	11	Specify the primary source of funding for the review.	Yes
Registration	12	Provide the register name and registration number.	Yes

From: Page MJ, McKenzie JE, Bossuyt PM, Boutron I, Hoffmann TC, Mulrow CD, et al. The PRISMA 2020 statement: an updated guideline for reporting systematic reviews. BMJ 2021;372:n71. doi: 10.1136/bmj.n71

For more information, visit: http://www.prisma-statement.org/

Supplementary Table 3 - PRISMA 2020 full checklist

PRISMA 2020 Checklist

Section and Topic	Item #	Checklist item	Location where item is reported
**TITLE**			
Title	1	Identify the report as a systematic review.	Page 1 line 1
**ABSTRACT**			
Abstract	2	See the PRISMA 2020 for Abstracts checklist.	Page 2
**INTRODUCTION**			
Rationale	3	Describe the rationale for the review in the context of existing knowledge.	Page 2-4 – "Introduction"
Objectives	4	Provide an explicit statement of the objective(s) or question(s) the review addresses.	Page 4 first paragraph
**METHODS**			
Eligibility criteria	5	Specify the inclusion and exclusion criteria for the review and how studies were grouped for the syntheses.	Page 17 – "Literature Selection"
Information sources	6	Specify all databases, registers, websites, organisations, reference lists and other sources searched or consulted to identify studies. Specify the date when each source was last searched or consulted.	Page 16 - "Literature Search"
Search strategy	7	Present the full search strategies for all databases, registers and websites, including any filters and limits used.	Supplementary Note 1
Selection process	8	Specify the methods used to decide whether a study met the inclusion criteria of the review, including how many reviewers screened each record and each report retrieved, whether they worked independently, and if applicable, details of automation tools used in the process.	Page 16-17 – "Literature Selection"
Data collection process	9	Specify the methods used to collect data from reports, including how many reviewers collected data from each report, whether they worked independently, any processes for obtaining or confirming data from study investigators, and if applicable, details of automation tools used in the process.	Page 19-20 - "Data Synthesis"
Data items	10a	List and define all outcomes for which data were sought. Specify whether all results that were compatible with each outcome domain in each study were sought (e.g. for all measures, time points, analyses), and if not, the methods used to decide which results to collect.	Page 19-20 – "Data Synthesis"
	10b	List and define all other variables for which data were sought (e.g. participant and intervention characteristics, funding sources). Describe any assumptions made about any missing or unclear information.	Supplementary Table 1
Study risk of bias assessment	11	Specify the methods used to assess risk of bias in the included studies, including details of the tool(s) used, how many reviewers assessed each study and whether they worked independently, and if applicable, details of automation tools used in the process.	Page 17-19 – "Risk of Bias assessment"
Effect measures	12	Specify for each outcome the effect measure(s) (e.g. risk ratio, mean difference) used in the synthesis or presentation of results.	Page 8-9 - "Analysis in Included Literature"
Synthesis methods	13a	Describe the processes used to decide which studies were eligible for each synthesis (e.g. tabulating the study intervention characteristics and comparing against the planned groups for each synthesis (item #5)).	Page 19-20 – "Data Synthesis"
	13b	Describe any methods required to prepare the data for presentation or synthesis, such as handling of missing summary statistics, or data conversions.	NA
	13c	Describe any methods used to tabulate or visually display results of individual studies and syntheses.	Page 19-20 – "Data Synthesis"
	13d	Describe any methods used to synthesize results and provide a rationale for the choice(s). If meta-analysis was performed, describe the model(s), method(s) to identify the presence and extent of statistical heterogeneity, and software package(s) used.	Page 19-20 – "Data Synthesis"
	13e	Describe any methods used to explore possible causes of heterogeneity among study results (e.g. subgroup analysis, meta-regression).	NA
	13f	Describe any sensitivity analyses conducted to assess robustness of the synthesized results.	NA
Reporting bias assessment	14	Describe any methods used to assess risk of bias due to missing results in a synthesis (arising from reporting biases).	NA
Certainty assessment	15	Describe any methods used to assess certainty (or confidence) in the body of evidence for an outcome.	NA

Section and Topic	Item #	Checklist item	Location where item is reported
**RESULTS**			
Study selection	16a	Describe the results of the search and selection process, from the number of records identified in the search to the number of studies included in the review, ideally using a flow diagram.	Page 4 – "Results" and Figure 1
	16b	Cite studies that might appear to meet the inclusion criteria, but which were excluded, and explain why they were excluded.	NA
Study characteristics	17	Cite each included study and present its characteristics.	Table 2
Risk of bias in studies	18	Present assessments of risk of bias for each included study.	Table 1
Results of individual studies	19	For all outcomes, present, for each study: (a) summary statistics for each group (where appropriate) and (b) an effect estimate and its precision (e.g. confidence/credible interval), ideally using structured tables or plots.	Table 3
Results of syntheses	20a	For each synthesis, briefly summarise the characteristics and risk of bias among contributing studies.	Page 4-9 – "Results"
	20b	Present results of all statistical syntheses conducted. If meta-analysis was done, present for each the summary estimate and its precision (e.g. confidence/credible interval) and measures of statistical heterogeneity. If comparing groups, describe the direction of the effect.	NA
	20c	Present results of all investigations of possible causes of heterogeneity among study results.	NA
	20d	Present results of all sensitivity analyses conducted to assess the robustness of the synthesized results.	NA
Reporting biases	21	Present assessments of risk of bias due to missing results (arising from reporting biases) for each synthesis assessed.	NA
Certainty of evidence	22	Present assessments of certainty (or confidence) in the body of evidence for each outcome assessed.	NA
**DISCUSSION**			
Discussion	23a	Provide a general interpretation of the results in the context of other evidence.	Page 10-15 – "Discussion"
	23b	Discuss any limitations of the evidence included in the review.	Page 13-15 "Current Limitations and Future Recommendations"
	23c	Discuss any limitations of the review processes used.	Page 12 – "Limitations of the Review"
	23d	Discuss implications of the results for practice, policy, and future research.	Page 13-15 "Current Limitations and Future Recommendations"
**OTHER INFORMATION**			
Registration and protocol	24a	Provide registration information for the review, including register name and registration number, or state that the review was not registered.	Page 16 – "Literature search"
	24b	Indicate where the review protocol can be accessed, or state that a protocol was not prepared.	Page 16 – "Literature search"
	24c	Describe and explain any amendments to information provided at registration or in the protocol.	Page 19 – "Data Synthesis"
Support	25	Describe sources of financial or non-financial support for the review, and the role of the funders or sponsors in the review.	Page 20 – "Acknowledgments"
Competing interests	26	Declare any competing interests of review authors.	Page 20 – "Competing Interests"
Availability of data, code and other materials	27	Report which of the following are publicly available and where they can be found: template data collection forms; data extracted from included studies; data used for all analyses; analytic code; any other materials used in the review.	Page 19-20 – "Data Synthesis" and "Data availability"

From: Page MJ, McKenzie JE, Bossuyt PM, Boutron I, Hoffmann TC, Mulrow CD, et al. The PRISMA 2020 statement: an updated guideline for reporting systematic reviews. BMJ 2021;372:n71. doi: 10.1136/bmj.n71

For more information, visit: http://www.prisma-statement.org/
